# Treatment of purely ligamentous dorsoulnar radiocarpal dislocation with ulnar translation of the carpus: A case report

**DOI:** 10.1097/MD.0000000000023451

**Published:** 2020-11-25

**Authors:** Il-Jung Park, Jongmin Kim, Jong Won Baek, Soo Hwan Kang

**Affiliations:** a Department of Orthopaedic Surgery, Bucheon St. Mary's Hospital; b Department of Orthopaedic Surgery, St. Vincent's Hospital, College of Medicine, The Catholic University of Korea, Seoul, Korea.

**Keywords:** purely ligamentous injuries, radiocarpal fracture-dislocation, ulnar translation of the carpus

## Abstract

**Rationale:**

Radiocarpal fracture-dislocations are extremely infrequent injuries caused by high-energy trauma and involve significant osseous and ligamentous injuries. If not treated properly, it can lead to serious complications such as ulnar translation of the carpus, multidirectional instability, loss of motion, and post-traumatic arthritis. Purely ligamentous injuries are rarer than fracture-dislocation injuries. Because previous studies have reported small patient cohorts, there has been no standardized treatment strategy for purely ligamentous radiocarpal dislocation.

**Patient concerns:**

A 24-year-old man suffered a left wrist injury in a motorcycle accident. Plain radiographs revealed dorso-ulnar radiocarpal dislocation without radial fracture and Carpal-ulnar distance ratio (CUDR) was 0.16. MRI scans showed the disruption of the dorsal ligaments and capsules and avulsed from the proximal insertion of the volar radiocarpal ligaments.

**Diagnosis:**

Dorsoulnar radiocarpal dislocation with purely ligamentous injury.

**Intervention:**

We removed the interposing chondral fragment from the radiocarpal joint and repaired the radioscaphocapitate (RSC) and radiolunate (RL) ligaments with the Jugger Knot Soft Anchor Suture (Biomet, Inc, Warsaw, IN) and applied additional radiocarpal K-wires and an external fixator to maintain reduction and optimal ligament tension.

**Outcomes:**

The patient showed good clinical results although ulnar translation of the carpus recurred in radiological follow-up.

**Lessons:**

Aggressive surgical management is needed earlier in the treatment of purely ligamentous radiocarpal dislocation, especially if the ulnar translation of the carpus was observed in the initial radiographs.

## 1 Introduction

Radiocarpal fracture-dislocation is a rare and complex injury; it represents about 0.2% of all dislocations. Common injury mechanisms include high-speed motor vehicle accidents, falling from a height, and industrial injury. Although dislocation can occur in any direction, dorsal dislocations occur most frequently. Dumontier et al^[1]^ classified radiocarpal fracture-dislocations into two groups. Group 1 included purely ligamentous injuries or fracture-dislocations associated with a small radial styloid avulsion; group 2 included radial styloid fractures involving at least one-third of the scaphoid fossa. Although many treatments have been described, the current treatment recommendation is surgical management. Goals of surgery include concentric reduction of the radiocarpal joint and osseous or soft tissue repair.^[2]^ Unfortunately, various complications including decreased wrist motion and recurrent radiocarpal dislocation or stiffness, have been reported in spite of the operation.^[1,2]^ Herein, we report the case of a 24-year-old man with purely ligamentous dorsoulnar radiocarpal dislocation who were treated by repair of volar radiocarpal ligaments and fixation with external fixator. In addition, we would like to report the result of our patient and other surgical methods through literatures review.

## 2 Case presentation

**Figure 1:**
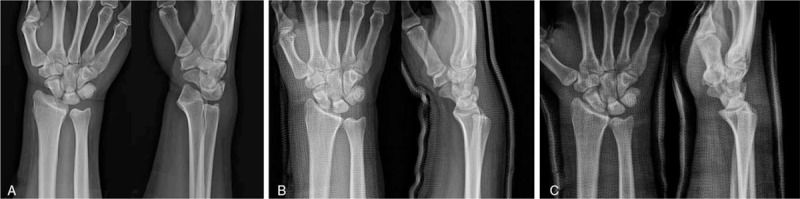
Preoperative plain radiographs. Carpal-ulnar distance ratio (CUDR) was used for measuring ulnar translation of the carpus. (A) Initial (CUDR = 0.16), (B) closed reduction (CUDR = 0.24), and (C) 3 weeks after the injury (CUDR = 0.16).

**Figure 2:**
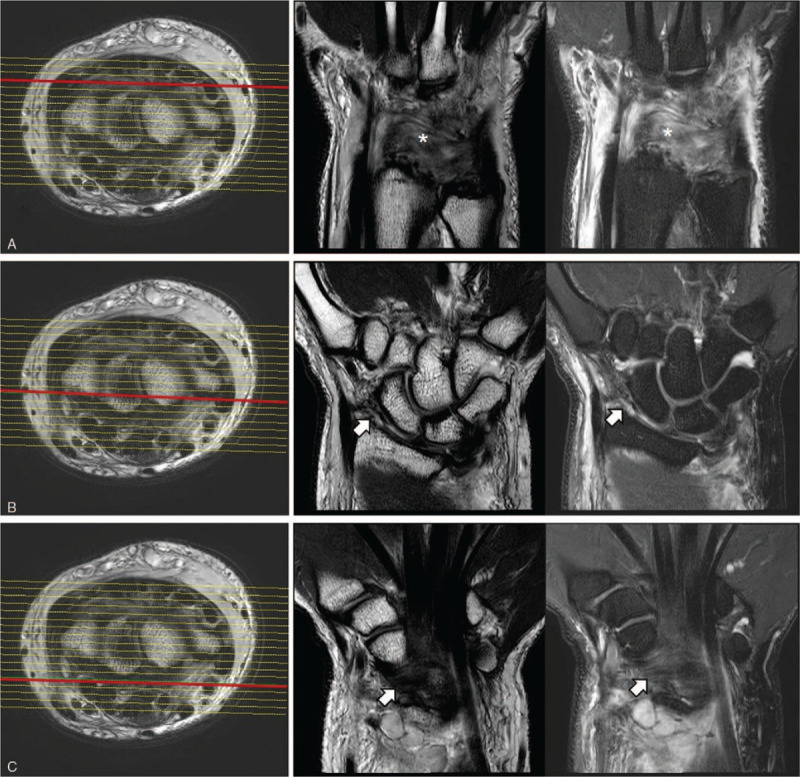
Preoperative MRI scans. (A) Disruption of the dorsal ligaments and capsules, (B and C) avulsed from the proximal insertion of the volar radiocarpal ligaments. MRI = magnetic resonance imaging.

**Figure 3:**
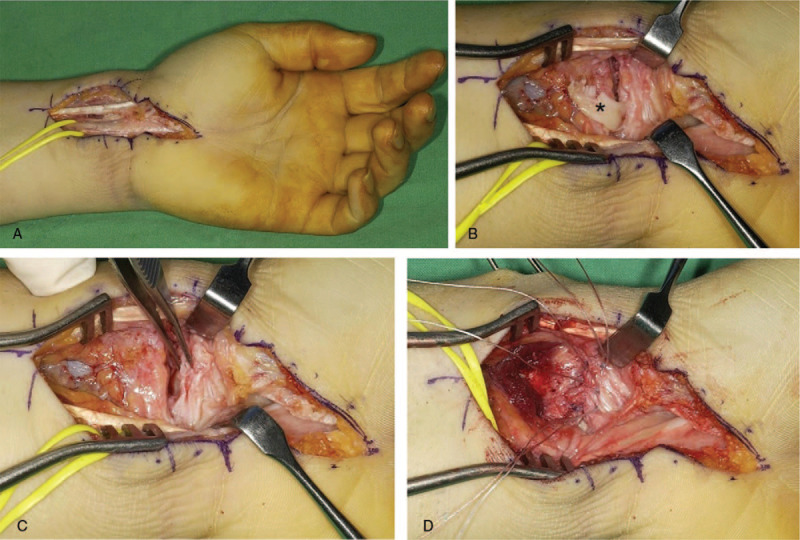
Intraoperative photographs. (A) Approximately 8 cm sized extensive palmar approach, (B) interposing chondral fragment (asterisk) from the radiocarpal joint, (C and D) repair of the RSC and RL ligaments with an anchor suture. RL = radiolunate, RSC = radioscaphocapitate.

**Figure 4:**
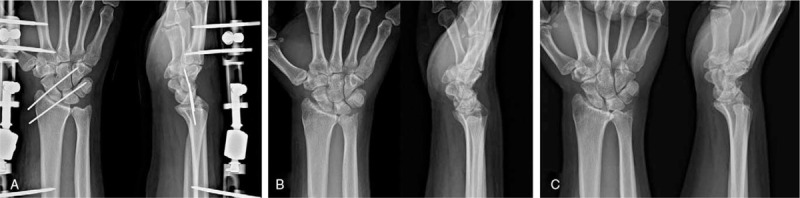
Postoperative plain radiographs. (A) Immediate postoperative (CUDR = 0.24), (B) 4 months postoperative (CUDR = 0.16), and (C) last follow-up at 22 months (CUDR = 0.18). CUDR = Carpal-ulnar distance ratio.

A 24-year-old man sustained an injury in a motorcycle accident. Plain radiographic findings revealed dorsoulnar radiocarpal dislocation without radial fracture. Carpal-ulnar distance ratio (CUDR) was used for measuring ulnar translation of the carpus. A line was drawn between the longitudinal axis of the ulna and the center of the capitate head, and the length of this line was divided by the third metacarpal length.^[3,4]^ The patient's CUDR was 0.16 at initial radiographs (Fig. 1A). The patient had a mild tingling sensation and hypesthesia along the median nerve dermatome. Our initial treatment was closed reduction and the radiocarpal joint reduced easily by manual traction. We performed magnetic resonance imaging (MRI) scans to examine the radius fracture and intrinsic carpal ligament injuries in greater detail. The MRI scans showed the disruption of the dorsal ligaments and capsules and avulsed from the proximal insertion of the volar radiocarpal ligaments (Fig. 2). Subsequently, the patient was diagnosed with dorsoulnar radiocarpal dislocation with a purely ligamentous injury. We decided to perform the conservative treatment with long arm casting because the radiocarpal joint looked well reduced after the closed reduction (CUDR = 0.24) (Fig. 1B). However, 3 weeks later after the injury, ulnar translation of the whole carpus progressed (CUDR = 0.16) (Fig. 1C), and therefore, we decided to perform the surgery. We selected an extensive palmar approach because the radioscaphocapitate (RSC) and radiolunate (RL) ligaments were the key structures to prevent ulnar translation of the carpus (Fig. 3A). An approximately 8 cm sized zig–zag skin incision was made. Dissection, including carpal tunnel release, was performed toward the radiocarpal joint. We could find that the RSC and RL ligaments were avulsed from the proximal insertion. We removed the interposing chondral fragment (asterisk) from the radiocarpal joint (Fig. 3B) and repaired the RSC and RL ligaments with the Jugger Knot Soft Anchor Suture (Biomet, Inc, Warsaw, IN) (Fig. 3C and D). We found the wrist to be stable. However, because the surgical repair was delayed due to initial conservative treatment, we decided to apply additional radiocarpal K-wires and an external fixator to maintain reduction and optimal ligament tension (CUDR = 0.24) (Fig. 4A).

**Figure 5:**
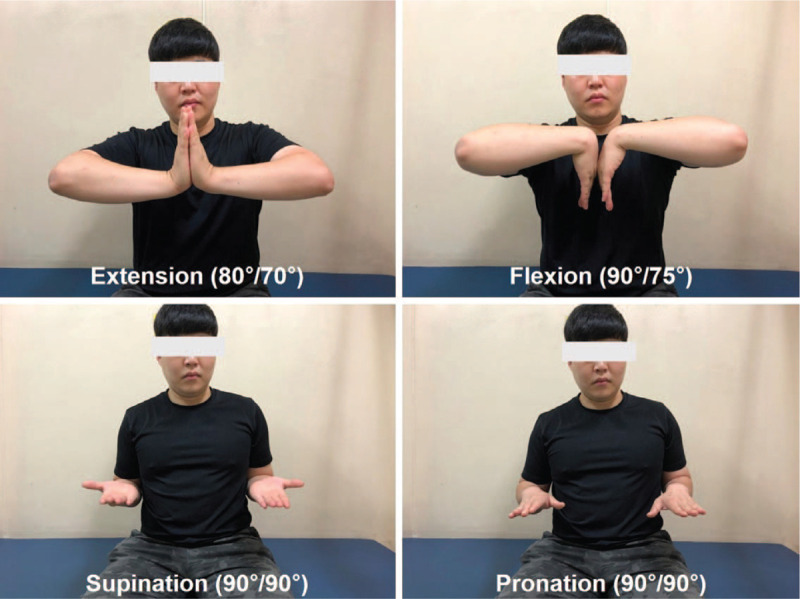
Clinical photographs of wrist range of motion at 22 months of follow-up.

After the K-wires and external fixator were removed at 8 weeks postoperative, the ulnar translation of carpal bone unfortunately recurred (CUDR = 0.16) (Fig. 4B). Outpatient follow-up was observed without further surgery. The patient's condition gradually improved, and the ulnar translation of the carpus was no longer progressing (Fig. 4C). At 22 months postoperative, the patient had no pain, nearly normal strength, and nearly full active range of motion without instability (CUDR = 0.18) (Fig. 5). The patient has no problem continuing his current job as a machine builder. The modified Mayo wrist score was 95 and the score on the Disabilities of the Arm, Shoulder and Hand questionnaire was 5 points.

## 3 Discussion

Radiocarpal fracture-dislocations are extremely infrequent injuries caused by high-energy trauma and involve significant osseous and ligamentous injuries. If not treated properly, they can lead to serious complications such as ulnar translation of the carpus, multidirectional instability, loss of motion, and post-traumatic arthritis.^[5]^

Purely ligamentous injuries are rarer and have inferior outcomes than fracture-dislocation injuries.^[1,2,6]^ Poor-quality tissue for repair leads to considerable challenges in maintaining reduction and preventing progressive ulnar translation of the carpus.^[1]^ Because previous studies have reported small patient cohorts, there has been no standardized treatment strategy for purely ligamentous radiocarpal dislocation. However, there have been three recommended treatment principles:

1. concentric reduction of the radiocarpal joint,
2. identification and treatment of intercarpal injuries, and
3. stable repair of the osseous-ligamentous avulsions.^[2]^

There were a few reports demonstrating that rigid immobilization was possible if stable and normal bony alignments were successfully restored after closed reductions.^[7]^ However, we consider these injuries to be very complex and unstable conditions that routinely warrant surgical reduction and fixation to attain a stable, concentric wrist. Therefore, several current treatment recommendations involve surgical management. Some authors have demonstrated that ulnar translation of the carpus is the predictable consequence of an avulsion injury to the volar radiocarpal ligaments, specifically the RSC and RL ligaments.^[1,8,9]^ Rayhack et al^[8]^ sequentially sectioned the radiocarpal ligaments and found that ulnar subluxation of the wrist joint required transection of both the RSC and RL ligaments. Both Dumontier et al^[1]^ and Yuan et al^[10]^ concluded that volar radiocarpal ligament repair leads to better outcomes. Therefore, we performed repair of the volar radiocarpal ligaments with an extensive palmar approach. During the operation, we found the wrist to be stable. However, because of the delayed surgical repair, we performed additional radiocarpal fixation and an external fixator to maintain reduction and optimal ligament tension. Contrary to our decision, Viegas et al^[11]^ reported in a cadaveric study that ulnar translation occurs when all extrinsic wrist ligaments are damaged. Therefore, repairing only the RSC and RL ligaments are not enough to prevent ulnar translation. Dumontier et al^[1]^ also reported that reduction was maintained after the volar and dorsal approaches were performed to repair the capsules and ligaments.

There are some reports on progressive ulnar translation of the carpus after the operation. Yuan et al^[10]^ reported that 3 patients out of 13 had progressive ulnar subluxation after the ligaments repair with radiographic follow-up, although there was no detailed information on the patient. Rayhack et al^[8]^ reported that all four patients treated with repair of the palmar ligaments had recurrent ulnar translation, and two required total wrist arthrodesis. Wahl et al^[9]^ reported there was only 1 case (8%) of ulnar carpal translation after treat the radiocarpal fracture-dislocation using dorsal wrist spanning plate fixation. In our study, this patient showed good clinical results, but the radiological reports showed that the ulnar translation recurred postoperatively. There are several possible reasons for this recurrence. First, the surgical repair was delayed due to initial conservative treatment. Second, because we only performed the volar approach, we could have been limited by the removal of interposed tissue from the radiocarpal joint. Finally, we did not restore the dorsal ligaments and capsules. Viegas et al^[11]^ suggested that the presence of ulnar translation represents a much more global ligament disruption. If the ulnar translation of the carpus continues in this patient, the radiocarpal ligament reconstruction using brachioradialis or limited wrist arthrodesis such as RL arthrodesis are viable treatment strategy.^[8,12]^

We concluded that aggressive surgical management was needed earlier in the treatment, especially if the ulnar translation of the carpus was observed in the initial radiographs. Additionally, we believe that if debris from the joint was removed and the dorsal and volar ligaments were well repaired, the results would have been better. And other alternative treatment methods such as ligament reconstruction may be considered instead of ligament repair because of the high risk of recurrence.

## Acknowledgments

We thank Chang Deok Weon, a medical photographer of Bucheon St. Mary's Hospital, the Catholic University of Korea for helping in preparing the photo.

## Author contributions

**Conceptualization:** Il-Jung Park, Soo Hwan Kang

**Data curations:** Il-Jung Park

**Supervision:** Soo Hwan Kang

**Validation:** Jong Won Baek

**Writing – original draft:** Jongmin Kim,

**Writing – review & editing:** Il-Jung Park
